# Domestic

**Published:** 1840-08-08

**Authors:** 


### DOMESTIC.

*A Phenomenon.*—Our readers will remember, that during this summer, the strange renewal of the eye of our fellow citizen, Captain George Davey, has been a subject of discussion in the newspapers. The statements which have been made are not in all particulars true. He has been appealed to, and gives the following statement, to be found in the letter of Mr. Handy. Captain Davey is one of our most respectable citizens, and the statement he has furnished of this extraordinary phenomenon may be implicitly relied on.

Dear Sir,—You may have noticed, in various papers, statements in relation to the *new eye* (as he calls it) of our worthy citizen Capt. George Davey. Capt. Davey has seen some of the statements and pronounces them to be inaccurate. He has given me the statement of the case, which I send you this day, and expresses a wish to have it published, that any errors in fact may be corrected; and that the exceeding singularity of the case may be made known. The case may give rise to valuable investigations of this delicate organ of the human system. Doubtless many will be the speculations pertaining to it. It is unnecessary to say here that Capt. Davey is a highly respectable, worthy, and intelligent man.

STATEMENT.

"In the year 1779—about the last of March, when I was about ten years and nine months old—an inflammation fell upon the eye-ball of my right eye and continued in a high degree for about six weeks, giving exceeding great pain during the whole time. At the expiration of six weeks, suppuration took place, and a discharge of corrupt pus. About the end of another six weeks, when the eye-ball was completely gone, and the socket sunken, my father discovered a *new eye*, resembling a bead or bird's eye. Strange as it may appear, the discovery was real; and the miniature eye grew until about the middle of July, when it attained its perfect size, and the vision was as clear and distinct as ever it had been, and so continued for eighteen months. At this time, however, I was taken with a second inflammation in *both eyes*, occasioned by bathing before I had entirely recovered from the small-pox. This produced a thick strong film over the *new* eye obstructing my visage in it for about fifty-eight years. For the last seven or eight years the film spoken of has appeared to decrease, until I can now distinguish different shades imperfectly. I can even now see tolerably well through a telescope or microscope; but it is remarkable that no spectacles, out of the great numbers I have tried, have ever afforded me any assistance. I use occasionally a thick convex glass, but with little aid. I am now about seventy-two years of age."

This is, almost verbatim, the very language of Capt. Davey, and is sent to you for publication for the reasons assigned above. Capt. Davey informs me that many medical gentlemen have seen his eye, amongst others Professor Granville S. Pattison.

Respectfully yours,

LEVIN HANDY.

*August* 5, 1840.

*Somerset* (*Md.*) *Herald.*

